# Polymorphism of the Transcription Factor 7-Like 2 Gene (TCF7L2) Interacts with Obesity on Type-2 Diabetes in the PREDIMED Study Emphasizing the Heterogeneity of Genetic Variants in Type-2 Diabetes Risk Prediction: Time for Obesity-Specific Genetic Risk Scores

**DOI:** 10.3390/nu8120793

**Published:** 2016-12-06

**Authors:** Dolores Corella, Oscar Coltell, Jose V. Sorlí, Ramón Estruch, Laura Quiles, Miguel Ángel Martínez-González, Jordi Salas-Salvadó, Olga Castañer, Fernando Arós, Manuel Ortega-Calvo, Lluís Serra-Majem, Enrique Gómez-Gracia, Olga Portolés, Miquel Fiol, Javier Díez Espino, Josep Basora, Montserrat Fitó, Emilio Ros, José M. Ordovás

**Affiliations:** 1 Department of Preventive Medicine and Public Health, School of Medicine, University of Valencia, 46010 Valencia, Spain; jose.sorli@uv.es (J.V.S.); laura.quiles@uv.es (L.Q.); olga.portoles@uv.es (O.P.); 2 CIBER Fisiopatología de la Obesidad y Nutrición, Instituto de Salud Carlos III, 28029 Madrid, Spain; oscar.coltell@uji.es (O.C.); RESTRUCH@clinic.cat (R.E.); mamartinez@unav.es (M.Á.M.-G.); jordi.salas@urv.cat (J.S.-S.); ocastaner@imim.es (O.C.); aborau@secardiologia.es (F.A.); 106mayorque104@gmail.com (M.O.-C.); lluis.serra@ulpgc.es (L.S.-M.); egomezgracia@uma.es (E.G.-G.); miguel.fiol@ssib.es (M.F.); javierdiezesp@ono.com (J.D.E.); jbasora.tarte.ics@gencat.cat (J.B.); MFito@imim.es (M.F.); EROS@clinic.ub.es (E.R.); jose.ordovas@tufts.edu (J.M.O.); 3 Department of Computer Languages and Systems, School of Technology and Experimental Sciences, Universitat Jaume I, 12071 Castellón, Spain; 4 Department of Internal Medicine, Hospital Clinic, IDIBAPS, 08036 Barcelona, Spain; 5 Department of Preventive Medicine and Public Health, University of Navarra—Navarra Institute for Health Research (IdisNa), 31009 Pamplona, Spain; 6 Human Nutrition Unit, Biochemistry and Biotechnology Department, IISPV, University Rovira i Virgili, 43003 Reus, Spain; 7 Cardiovascular Risk and Nutrition Research Group, Hospital del Mar Medical Research Institute (IMIM), 08003 Barcelona, Spain; 8 Department of Cardiology, Hospital Txagorritxu, 01009 Vitoria, Spain; 9 Department of Family Medicine, Distrito Sanitario Atención Primaria Sevilla, Centro de Salud Las Palmeritas, 41003 Sevilla, Spain; 10 Research Institute of Biomedical and Health Sciences, University of Las Palmas de Gran Canaria, 35001 Las Palmas de Gran Canaria, Spain; 11 Department of Epidemiology, School of Medicine, University of Malaga, 29071 Malaga, Spain; 12 Palma Institute of Health Research (IdISPa), Hospital Son Espases, 07014 Palma de Mallorca, Spain; 13 Department of Preventive Medicine and Public Health, University of Navarra—Navarra Institute for Health Research (IdisNA)—Servicio Navarro de Salud-Osasunbidea, 31009 Pamplona, Spain; 14 Lipid Clinic, Endocrinology and Nutrition Service, Institut d’Investigacions Biomèdiques August Pi Sunyer (IDIBAPS), Hospital Clinic, 08036 Barcelona, Spain; 15 Nutrition and Genomics Laboratory, JM-USDA Human Nutrition Research Center on Aging at Tufts University, Boston, MA 02111, USA; 16 Department of Cardiovascular Epidemiology and Population Genetics, Centro Nacional de Investigaciones Cardiovasculares (CNIC), Madrid 28029—IMDEA Alimentación, 28049 Madrid, Spain

**Keywords:** TCF7L2, type-2 diabetes, obesity, T2D-genetic risk scores, TCF7L2-predictive value, PREDIMED study

## Abstract

Nutrigenetic studies analyzing gene–diet interactions of the TCF7L2-rs7903146 C > T polymorphism on type-2 diabetes (T2D) have shown controversial results. A reason contributing to this may be the additional modulation by obesity. Moreover, TCF7L2-rs7903146 is one of the most influential variants in T2D-genetic risk scores (GRS). Therefore, to increase the predictive value (PV) of GRS it is necessary to first see whether the included polymorphisms have heterogeneous effects. We comprehensively investigated gene-obesity interactions between the TCF7L2-rs7903146 C > T polymorphism on T2D (prevalence and incidence) and analyzed other T2D-polymorphisms in a sub-sample. We studied 7018 PREDIMED participants at baseline and longitudinally (8.7 years maximum follow-up). Obesity significantly interacted with the TCF7L2-rs7903146 on T2D prevalence, associations being greater in non-obese subjects. Accordingly, we prospectively observed in non-T2D subjects (*n* = 3607) that its association with T2D incidence was stronger in non-obese (HR: 1.81; 95% CI: 1.13–2.92, *p* = 0.013 for TT versus CC) than in obese subjects (HR: 1.01; 95% CI: 0.61–1.66; *p* = 0.979; *p*-interaction = 0.048). Accordingly, TCF7L2-PV was higher in non-obese subjects. Additionally, we created obesity-specific GRS with ten T2D-polymorphisms and demonstrated for the first time their higher strata-specific PV. In conclusion, we provide strong evidence supporting the need for considering obesity when analyzing the TCF7L2 effects and propose the use of obesity-specific GRS for T2D.

## 1. Introduction

It is common knowledge that obesity is associated with an increased risk of developing type 2 diabetes (T2D) [1,2,3]. However, current genetic information adds some heterogeneity to this notion [4]. Thus, whereas some genetic variants may appear to be associated with T2D mainly in obese subjects [5,6,7], others may show such association primarily in non-obese individuals [5,6,8]. Understanding these differences is crucial to improving the predictive value of genetic variants when investigating T2D as well as gene–diet interactions. Currently, the rs7903146 C > T Single nucleotide polymorphism (SNP) in the Transcription Factor 7-Like 2 (TCF7L2) gene is the locus most strongly associated with T2D risk at the population level [9,10,11]. However, despite the strong overall association of this SNP with higher T2D risk, various studies have suggested a modulation of this association by obesity [6,12,13,14,15].

Cauchi et al. [6] first reported that the association between the TCF7L2-rs7903146 SNP and prevalent T2D in Europeans was stronger in non-obese subjects. These findings were observed in other populations [12,13,14,15]. Nevertheless, this potential heterogeneity by obesity has not been widely reflected in the analytical approaches of subsequent investigations, and most of them have not formally tested the interaction between the TCF7L2-rs7903146 polymorphism and obesity status in determining T2D risk. A contributory factor is that previous findings were mainly based on cross-sectional or case-control studies [5,6,9,12,13,14] with a strong likelihood of being affected by potential biases, more prospective studies being required to assess this interaction on T2D incidence. Moreover, in addition to the TCF7L-2-rs7903146 SNP, other SNPs have been associated with T2D risk [10,16,17,18]. These SNPs are combined and analyzed together in the so-called genetic risk scores (GRS) to predict T2D [16,17,18]. However, simply summing up the number of risk alleles (unweighted or weighted) associated with T2D obtained from non-stratified genome-wide association studies (GWAS) in conventional GRS calculations may overlook important obesity-specific associations in T2D. Although the GRS usually include dozens of SNPs associated with T2D, one of the most important SNPs is the rs7903146 C > T in the TCF7L2 gene [9,10,11,16]. Recently, large prospective studies have focused on the interaction between some multi-SNP GRS and BMI on T2D incidence [16,17,18,19], among them, that of Langerberg et al. [16], employing a case-cohort design in the EPIC interact study. The authors found a statistically significant interaction between a GRS comprising 49 SNPs associated with T2D and BMI (three categories) in determining T2D incidence, the genetic risk being greater in lean subjects. However, on examining the interaction of each SNP of the GRS with BMI on T2D incidence, no statistically significant interaction with BMI was found for the TCF7L2-rs7903146 SNP [16]. This could be because they did not specifically test the interaction with obesity and made a strict correction for multiple comparisons due to the simultaneous analyses of 7 phenotypes and 49 SNPs in the same study. Bearing the results of the result in EPIC cohort in mind, this interaction, therefore, must be prospectively validated in studies focusing on the TCF7L2-rs7903146 polymorphism (to avoid the need of correction for multiple SNP comparisons) and obesity.

Furthermore, the heterogeneity of associations related to this locus also extends to BMI. The T-allele, conferring higher T2D risk, has been associated with lower BMI in some studies [20,21,22,23], but not in others [24,25,26]. A modulation of this association by T2D was first suggested by Helgason et al. [27] who showed that the TCF7L2-T2D risk allele was correlated with decreased BMI in T2D cases but not in controls. Similar results were observed both by Cauchi et al. [21] and in a meta-analysis including more than 300,000 individuals [28], but further studies are required to explore this interaction prospectively. Moreover, as previous findings come from studies focusing on either obesity or T2D, it is necessary to obtain comprehensive evidence of the interplay between both interactions prospectively in the same population. Therefore, our main aims were: (1) To investigate the interaction between the TCF7L2-rs7903146 polymorphism and obesity status in determining T2D prevalence as well as T2D incidence after a median ~6-year follow-up and (2) to examine whether the association of the TCF7L2-rs7903146 SNP with obesity-related parameters depends on T2D status both at baseline and prospectively in the PREvención con DIeta MEDiterránea (PREDIMED) study. In addition, a secondary aim was to construct obesity-specific GRS (analyzing 10 T2D-SNPs previously characterized [16]) in determining T2D prevalence in a sub-sample of PREDIMED participants in order to extend the findings to other T2D-SNPs.

## 2. Materials and Methods

The present study was conducted within the framework of the PREDIMED trial, the design of which has been described in detail elsewhere [29]. Briefly, the PREDIMED study is a multicenter, randomized, and controlled clinical trial aimed at assessing the effects of the Mediterranean diet (MedDiet) on the primary cardiovascular prevention [30]. This study was registered at controlled-trials.com (http://www.controlledtrials.com/ISRCTN35739639). Here we included 7018 participants from whom DNA was isolated, the TCF7L2-rs7903146 determined, and who had valid data for the main clinical and lifestyle variables analyzed. From October 2003 physicians in Primary Care Centers selected high cardiovascular risk participants. Eligible were community-dwelling persons (55–80 years for men; 60–80 years for women) who met at least one of two criteria: T2D or three or more cardiovascular risk factors [29]. The Institutional Review Board of each participating center approved the study protocol, and all participants provided written informed consent. The trial was stopped following the statistical analysis of data obtained up to December 2010 (median follow-up of 4.8 years), due to early evidence of the benefit of the MedDiet on the prevention of major cardiovascular events [30]. However, the ascertainment of endpoints was extended. The present study is based on the extended follow-up (until 30 June 2012) using the same methods to obtain updated information on clinical events, including T2D. The median follow-up time in this extended follow-up was 5.7 years (maximum: 8.7 years). The present study was mainly conducted as an observational prospective cohort design with adjustment for the nutritional intervention in the longitudinal analyses. In addition, some association analyses were carried out at baseline.

### 2.1. Demographic, Clinical, Anthropometric, and Dietary Measurements

The baseline examination included assessment of standard cardiovascular risk factors, medication use, socio-demographic factors, and lifestyle variables by validated questionnaires [29,30]. Weight and height were measured with calibrated scales and a wall-mounted stadiometer, respectively. BMI and the waist-to-height ratio were calculated. Obesity was defined as BMI ≥30 kg/m^2^. Percentage of body fat was evaluated by using a validated equation [31].

### 2.2. Biochemical Determinations, DNA Extraction and Genotyping

At baseline, blood samples were obtained after overnight fasting. Fasting glucose and lipids were measured as previously described [30,32]. Genomic DNA was extracted from buffy-coat and the TCF7L2-rs7903146, was genotyped in the whole cohort on a 7900 HT Sequence Detection System (Applied Biosystems, Foster City, CA, USA) using a fluorescent allelic discrimination TaqManTM assay as previously reported [33]. Genotype frequencies did not deviate from Hardy–Weinberg equilibrium expectations.

For the secondary outcome focused on the predictive value of the obesity-specific GRSs, in addition to the TCF7L2-rs7903146 SNP, nine previously described SNPs associated with T2D, and included in a 49-SNP T2D-GRS [16], were selected and genotyped. The selected SNPs were: PRC1 (Protein Regulator of Cytokinesis 1)-rs12899811, ZFAND6 (Zinc Finger AN1-Type Containing 6)-rs11634397, CDC123_CAMK1D (Cell Division Cycle Protein 123 Homolog_Calcium/Calmodulin Dependent Protein Kinase ID)-rs11257655, KCNQ1 (Potassium Voltage-Gated Channel Subfamily Q Member 1)-rs163184, ADYC5 (adenylyl cyclase 6)-rs6798189, IGF2BP2 (Insulin Like Growth Factor 2 MRNA Binding Protein 2)-rs4402960, SLC30A8 (Solute Carrier Family 30 Member 8)-rs3802177, KLHDC5 (Kelch Domain-Containing Protein 5)-rs10842994, and HMGA2 (High Mobility Group AT-Hook 2)-rs2261181. Genotyping was carried out with the HumanOmniExpress Illumina array in a sub-sample (all the participants from one of the PREDIMED field centers, the PREDIMED-Valencia center; *n* = 1055 subjects), as it was not possible to genotype the whole cohort. Genotype frequencies did not deviate from Hardy–Weinberg equilibrium expectations.

### 2.3. Outcomes and Follow-Up

Clinical diagnosis of T2D was an inclusion criterion of the PREDIMED study [29], and these subjects were considered as prevalent cases of T2D. Incidence of T2D was a pre-specified secondary outcome of the PREDIMED trial [30]. New-onset diabetes during follow-up was diagnosed using the American Diabetes Association criteria, namely fasting plasma glucose levels ≥7.0 mmol/L (≥126.1 mg/dL) or 2-h plasma glucose levels ≥11.1 mmol/L (≥200.0 mg/dL) after a 75-g oral glucose load, as previously reported [32]. A review of all medical records of participants was completed yearly in each center by physician-investigators who were blinded to the intervention. When new-onset diabetes cases were identified on the basis of a medical diagnosis reported in the medical charts or on a glucose test during routine biochemical analyses (conducted at least once per year), these reports were sent to the PREDIMED Clinical Events Committee [32]. When a new case of T2D was detected, the glucose analysis was repeated within the next three months, so that the new case of diabetes could be confirmed by the adjudication committee. Cases that occurred between 1 October 2003 and 30 June 2012 (maximum: 8.7 years; median: 5.7 years) were included in the present analysis (*n* = 312).

Given that the study involved an open cohort, in which the inclusion of participants lasted from 1 October 2003 to 1 December 2009, not all participants had the same length of follow-up period [29]. Hence for the longitudinal analyses of BMI in relation to the polymorphism and T2D, two follow-up periods were selected; one of up to four years and the other up to six years. There were a greater number of participants in the first period (*n* = 3141), as most of the cohort completed this follow-up period. A lower number of participants had anthropometric measurements at six years (*n* = 1750), but this group was considered to be of interest both for the internal replication of the finding and for providing more evidence of the interaction. Only participants whose anthropometric data had been directly measured were included.

### 2.4. Statistical Analyses

The present analysis was mainly conducted as an observational prospective cohort study with adjustment for the nutritional intervention in longitudinal analyses. In addition, some analyses were carried out cross-sectionally at baseline (*n* = 7018). Prevalence of diagnosed T2D was analyzed as the dependent variable at baseline. In the longitudinal analysis, incidence of T2D was considered as the end-point in non-diabetic subjects (*n* = 3607). Moreover, baseline and annual BMI evolution was considered as the dependent variable for evaluating the interaction of the polymorphism with T2D in determining BMI.

#### 2.4.1. Baseline Association and Interaction Analyses in Determining T2D Prevalence and Obesity-Related Variables

Chi-square tests were used to test differences in percentages. We first tested the polymorphism by considering the 3 genotypes. The interactions between the TCF7L2-rs7903146 polymorphism and obesity in determining T2D prevalence at baseline was tested by multivariable logistic regression models including main effect and interaction terms. Models were adjusted for basic potential confounders (age, gender, and center) (Model 1). Afterwards, an additional control for more potential confounders such as alcohol consumption, physical activity, adherence to the MedDiet, total energy intake, hypertension, and dyslipidemias was undertaken (Model 2). Analyses stratified by obesity status were also undertaken for models 1 and 2. CC subjects were considered as the reference category and the effect in CT and TT was estimated. Odds ratios (OR) and 95% Confidence intervals (CI) were estimated. Likewise, the interaction between the TCF7L2 polymorphism and T2D in determining obesity prevalence at baseline was evaluated by multivariable logistic regression models (model 1 and model 2), and stratified analysis by T2D status undertaken. In addition, associations between the TCF7L2 polymorphism and baseline BMI and other obesity-related variables were analyzed by linear regression models including main effects and interaction terms. Multivariable adjustments for potential confounding variables were carried out as indicated above. Analyses stratified by T2D were also undertaken.

#### 2.4.2. Interaction Analysis between the TCF7L2-rs7903146 Polymorphism and Obesity in Determining T2D Incidence

This analysis was carried out in non-T2D subjects at baseline. We used Cox regression models with the length of follow-up as the primary time variable. Follow-up time was calculated from the date of enrollment to the date of diagnosis of T2D for cases, and to the date of the last visit or the end of the follow-up period (30 June 2012 for non-cases), or the date at death, whichever came first. Hazard ratios (HR) with 95% CI for the TCF7L2-rs7903146 genotypes (three categories), stratified by obesity were computed. Afterwards, C-allele carriers were grouped together and compared with C-carriers (recessive model). Multivariable Cox regression models with main effects and interaction terms were computed. In multivariable Model 1 (basic model) we adjusted for sex, age, center, and intervention group. In multivariable Model 2 additional adjustments were undertaken as previously described. Stratified analyses by obesity were carried out. In addition, Kaplan–Meier survival curves (one minus the cumulative T2D free survival) were plotted to estimate the probability of remaining free of T2D during follow-up depending on the TCF7L2 genotype and obesity status.

#### 2.4.3. Predictive Value Calculations for the TCF72-rs7903146 Polymorphism on T2D Incidence and Prevalence in the Whole PREDIMED Participants

To estimate the predictive ability of the genetic models depending on the obesity status, we used two approaches: (a) In non-T2D subjects, we estimated its sensitivity, specificity, positive predictive value (PPV), negative predictive value (NPV) for two categories (recessive model) in predicting T2D incidence taking into account obesity status; (b) At baseline, we estimated the area under the receiver operating characteristic curve (AUC) [19] of the TCF7L2-rs7903146 (as 0, 1 and 2) to predict T2D prevalence depending on obesity status (we selected the recessive and additive models for T2D incidence and prevalence prediction based on the observed association results).

#### 2.4.4. Construction of Obesity-Specific GRS with the TCF7L2 and Other T2D-SNPs; Association and Evaluation of the PV for T2D Prevalence

Taking into account the obesity-specific association of the TCF7L2 polymorphism with T2D, our secondary aim was to extend this analysis to more T2D SNPs. This was considered a pilot study as we only have genotype data from one of the PREDIMED field centers (PREDIMED-Valencia participants with complete data; *n* = 1000 participants; 46% T2D prevalence). These SNPs were selected from the list of 49 SNPs associated with T2D that were used in the EPIC-InterAct study for the multi-SNP GRS construction and T2D association [16]. From the list of the 49 T2D-SNP, in addition to the TCF7L2-SNP, we selected those included in our genotyping array (*n* = 27) and specifically tested the association between the corresponding SNP and T2D by obesity status. Those SNPs showing suggestive heterogeneity in the associations in our population were included in the obesity-specific GRS analyses (PRC1-rs12899811, ZFAND6-rs11634397, CDC123_CAMK1D-rs11257655, KCNQ1-rs163184, ADYC5-rs6798189, IGF2BP2-rs4402960, SLC30A8-rs3802177, KLHDC5-rs10842994, and HMGA2-rs2261181). For some of these SNPs (in the ZFAND6, ADYC5, IGF2BP2, SLC30A8, KLHDC5, and HMGA2 genes), statistically significant or borderline significant interactions with BMI (or with waist circumference) in determining T2D were reported in the EPIC-InterAct study (*p* = 0.055, *p* < 0.01; *p* = 0.034; *p* = 0.099; *p* = 0.10, respectively) [16]. However, the authors did not construct obesity-specific GRS. Depending on the results obtained in the stratified analysis, SNPs were grouped in two obesity-specific GRS. One GRS included five T2D-SNPs more associated with T2D in obese subjects (obGRS); and the other GRS included five T2D-SNPs more associated with T2D in non-obese subjects (nobGRS). nobGRS: TCF7L2-rs7903146, PRC1-rs12899811, ZFAND6-rs11634397, CDC123_CAMK1D-rs11257655 and KCNQ1-rs163184; obGRS: ADYC5-rs6798189, IGF2BP2-rs4402960, SLC30A8-rs3802177, KLHDC5-rs10842994 and HMGA2-rs2261181. SNPs in these GRS were considered as additive (0, 1, or 2 risk alleles). Multivariable logistic regression models with prevalent T2D as dependent variable and the obesity-specific GRS (as continuous) as independent variables, adjusted for age, sex, and obesity were fitted for the total and for obese and non-obese subjects; OR and 95% CI were calculated to estimate the association between the GRS and T2D.

Finally; the AUC of the two GRS predicting T2D at baseline in the PREDIMED-Valencia subsample by obesity status (obesity-specific GRS) in the whole population and in obese and non-obese subjects were calculated.

#### 2.4.5. Longitudinal Association and Interaction Analysis between the TCF7L2-rs7903146 Polymorphism and T2D in Determining BMI

The longitudinal influence of the TCF7L2-rs7903146 polymorphism and T2D on BMI was analyzed by multivariable-ANCOVA of repeated measures including those subjects having complete data at baseline, 1, 2, 3, and 4 years (first four-year period) and at baseline, 1, 2, 3, 4, 5, and 6 years (second six-year period).

#### 2.4.6. Power Calculations

Sample size in the PREDIMED study (*n* = 7447 participants) was estimated taking into account the expected incidence of the primary outcome (incidence of cardiovascular diseases) and the differences in the effects of the dietary interventions to be detected among groups [30]. In the present study, we focused on T2D prevalence and T2D incidence in PREDIMED participants with the TCF7L2-*rs7903146* data available (*n* = 7018). At baseline our study (including *n* = 3607 non-T2D and *n* = 3411 T2D subjects), had a large statistical power (>80%) to detect associations (OR >1.2) at alpha = 5% between the TCF7L2 polymorphism and T2D prevalence in obese and non-obese subjects. Taking into account the similar sample size of T2D and non-T2D subjects at baseline, as well as the % of obese and non-obese subjects, our study has the strong advantage of having comparable statistical power to detect a similar association in both groups. Therefore, the lack of association between the TCF7L2-rs7903146 polymorphism and T2D risk in the stratified analyses in obese or non-obese subjects is not due to the lack of power in one of the groups. At baseline, our sample size was adequately powered (power >80%) to detect statistically significant TCF7L2-obesity interactions (at alpha <5%) in determining T2D prevalence (>40%) at an interaction effect of OR for interaction >1.21 (co-dominant model). Similar estimations in sample size and effects were computed for the interaction between the TC7L2 polymorphism and T2D in determining obesity risk. For continuous variables our sample size at baseline was adequately powered (power >80%) to detect statistically significant interactions and associations in the effect strata. In the longitudinal analysis, taking into account that the number of incident cases of T2D was small (*n* = 312) and that only non-T2D subjects at baseline were considered (*n* = 3607), the power to detect statistically significant interactions and association was lower than in the baseline analysis. Therefore, at alpha = 5% and beta = 20%, our sample size was adequately powered (>80%) to detected interaction effects (recessive model) of HR >1.75. Statistical analyses were performed with the IBM SPSS Statistics version 22, NY. All tests were two-tailed and *p* values < 0.05 were considered statistically significant.

## 3. Results

Table 1 shows the characteristics of the studied population (*n* = 7018 subjects) as a whole and depending on the T2D status at baseline.

### 3.1. Interaction between the TCF7L2-rs7903146 Polymorphism and Obesity in Determining T2D at Baseline

Even though the TCF7L2-rs7903146 polymorphism was significantly associated with a higher T2D prevalence in the whole population (*p* = 3.1 × 10^−21^), this association was of greater magnitude (OR: 2.26; 95% CI: 1.84–2.78 for TT compared to CC homozygotes; *p* = 1.6 × 10^−14^) in non-obese (*n* = 3739) than in obese (*n* = 3279) subjects (OR: 1.51; 95% CI: 1.22–1.92 for TT compared to CC homozygotes; *p* = 0.0002) even after multivariable adjustment (Table 2).

### 3.2. Interaction between the TCF7L2-rs7903146 Polymorphism and T2D in Determining Obesity-Related Measures at Baseline

**Figure 1:**
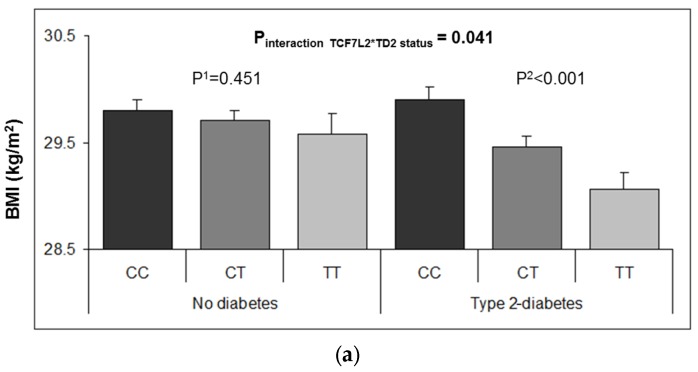

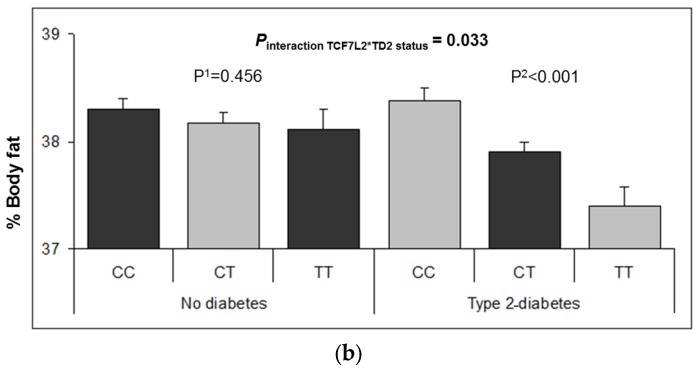
Adjusted means of BMI (**a**) and % body fat (**b**) at baseline depending on the TCF7L2-rs7903146 polymorphism and T2D diabetes status (*n* = in 7018 PREDIMED participants at baseline. Means were adjusted for age, sex, center, total energy intake, physical activity, smoking, drinking, adherence to the Mediterranean diet, dyslipidemia, and hypertension. The *p*-values for the interaction terms were obtained in the corresponding multivariable adjusted models. *p* ^1^ and *p* ^2^ values were obtained for the multivariable comparison of means between depending on the T2D strata in non-diabetic (*n* = 3607) subjects and T2D (*n* = 3411) subjects, respectively. Error bars: SE of means.

At baseline, the T2D risk allele was significantly associated with lower averages for BMI, body fat, waist-to-height ratio, and lower prevalence of obesity (BMI ≥30 kg/m^2^) (Supplementary Materials Table S1). However, we detected a strong heterogeneity in these associations depending on T2D status. Figure 1 shows these interactive effects on BMI (A) and % body fat (B), the TCF7L2-rs7903146 polymorphism being inversely associated with these traits only in T2D subjects (*p*-interactions < 0.05). Likewise, the inverse association between the TCF7L2-rs7903146 polymorphism and obesity at baseline (Table 3) was statistically significant in T2D (*p* = 0.0002) but not in non-T2D subjects (*p* = 0.737). Accordingly, the multivariable adjusted OR for obesity in TT subjects compared to CC individuals was 0.63; 95% CI 0.51–0.78; *p* = 1.63 × 10^−5^ in T2D, showing a reduced risk, and no association (0.93; 95% CI: 0.74–1.16; *p* = 0.493) in non-diabetic subjects.

### 3.3. Interaction between the TCF7L2-rs7903146 Polymorphism and Obesity in the Incidence of T2D

**Figure 2:**
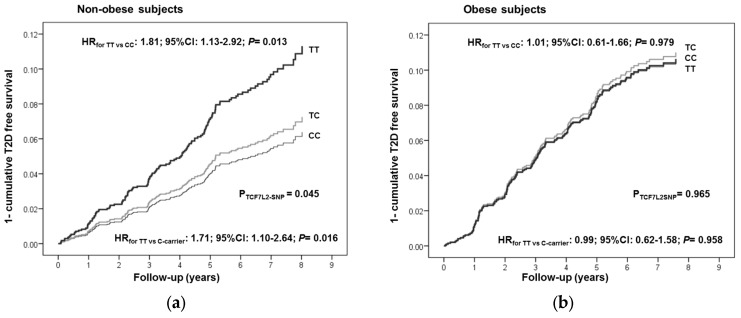
One minus the cumulative T2D-free survival by TCF7L2-rs7903146 genotypes in non-diabetic subjects at baseline (*n* = 3607) depending of the obesity status: non-obese subjects (**a**) and obese subjects (**b**). Cox regression models with outcome of T2D incidence by the TCF7L2-rs7903146 polymorphism (CC, CT and TT) were adjusted for sex, age, center, intervention group, alcohol, smoking, total energy intake and adherence to the Mediterranean diet, physical activity, smoking, drinking, dyslipidemia, and hypertension at baseline. HR and 95% CI were obtained in the multivariable adjusted model. The *p*-values for the TCF7L2 polymorphism and for the corresponding genotypes (TT versus CC or TT versus C-carriers) were obtained in the multivariable adjusted models.

We analyzed the interaction between the TCF7L2-rs7903146 polymorphism and obesity status in determining the incidence of T2D in non-diabetic subjects (*n* = 3607) over the extended follow-up period (with median 5.7 years) (Table 4). We observed that the association between the TCF7L2-rs7903146 and T2D incidence only was statistically significant in obese subjects (*p* = 0.035 in the basic model and *p* = 0.045 in the additionally adjusted model). Thus, we observer a significantly stronger association in non-obese (HR: 1.81; 95% CI: 1.13–2.92, *p* = 0.013 for TT versus CC) than in obese individuals (HR: 1.01; 95% CI: 0.61–1.66; *p* = 0.979); *p*-interaction = 0.048. A recessive effect was found in agreement with results obtained in previous studies [20]. Figure 2 shows T2D-free survival Kaplan–Meier curves in non-obese (A) and obese subjects (B). Taking into account that a recessive effect was noted, homozygous subjects for the risk allele (TT) were also compared with C-allele carriers.

Furthermore, we estimated the influence of obesity status on T2D incidence over the 5.7-year median follow-up period in this population in the whole sample and depending on the TCF7L2-rs7903146 polymorphism. Although for the whole population of T2D-free subjects at baseline obesity was significantly associated with higher TD2 risk (HR: 1.44; 95% CI: 1.14–1.82; *p* = 0.002), this association was heterogeneous in the different genotypes (Supplementary Materials Figure S1). Thus, in CC subjects, obesity was strongly associated with T2D incidence (HR: 1.75; 95% CI: 1.22–2.51; *p* = 0.002 in obese compared with non-obese). This significant association decreased in CT subjects (HR: 1.45; 95% CI: 102–2.07; *p* = 0.041 in obese versus non-obese subjects). No significant association between obesity status and T2D incidence was found in subjects with the TT genotype (HR: 0.79; 95% CI: 0.43–1.46; *p* = 0.454).

### 3.4. Predictive Ability of the TCF7L2-rs7903146 on T2D Incidence and Prevalence Depending on Obesity Status

Supplementary Materials Table S2 shows the sensitivity, specificity, PPV, and NPV for the TCF7L2-SNP (as recessive according to the observed association effect) on predicting T2D incidence in non-TD2 PREDIMED participants. Significantly better parameters were obtained in non-obese subjects (*p* = 0.03) than in obese subjects (*p* = 0.787).

Likewise, in the ROC analysis, the AUC for the TCF7L2 SNP (additive model) for T2D prevalence in PREDIMED participants was higher in non-obese (AUC: 0.58; *p* = 1.37 × 10^−17^) than in obese subjects (AUC: 0.53; *p* = 1.4 × 10^−4^) (Supplementary Materials Figure S2).

### 3.5. Obesity Specific-GRS Construction, Association with T2D Prevalence and Estimations of the Predictive Value of These GRS in a Subsample of Participants

**Figure 3:**
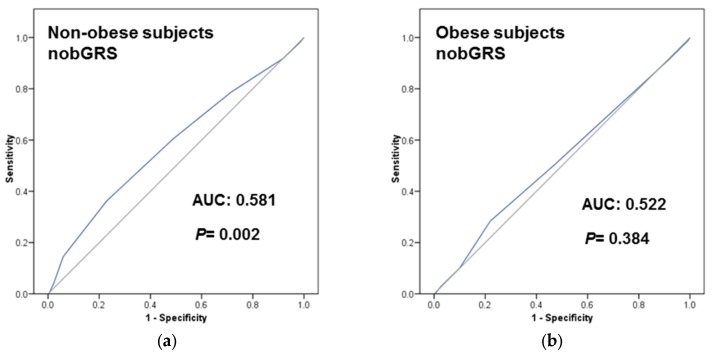

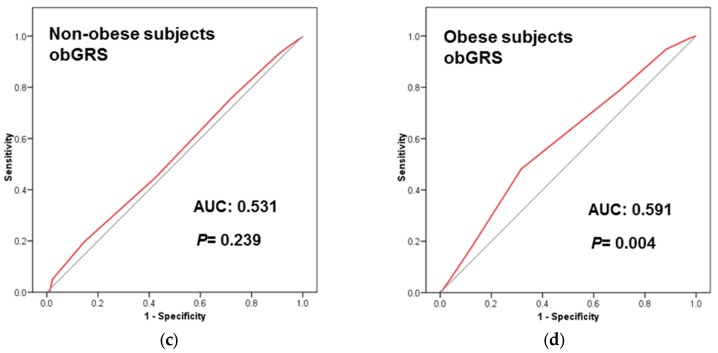
Receiver operating curves (ROC) of the two Genetic Risk Scores (GRS) [One including T2D-SNPs more associated in obese subjects (obGRS); and the other including T2D-SNPs more associated in non-obese subjects (nobGRS)] to predict T2D (prevalent) at baseline in the PREDIMED-Valencia participants (*n* = 1000): (**a**) nobGRS in non-obese subjects; (**b**) nobGRS in obese subjects; (**c**) obGRS in non-obese subjects; (**d**) obGRS in obese subjects. Areas under the curves (AUC) and *p*-values are indicated. The straight line represents the ROC expected by chance only. *n* = 493 non-obese and *n* = 507 obese with genotype data for all the SNPs included in the GRS were analyzed. Five SNPs were included in each unweighted additive (risk allele) GRS as follows: nobGRS: TCF7L2-rs7903146, PRC1-rs12899811, ZFAND6-rs11634397, CDC123-CAMK1D-rs11257655 and KCNQ1-rs163184; obGRS: ADYC5-rs6798189, IGF2BP2-rs4402960, SLC30A8-rs3802177, KLHDC5-rs10842994, and HMGA2-rs2261181.

Our secondary aim was to extend the TCF7L2 analysis to more T2D SNPs. This was considered a pilot study as we only have genotype data from one of the PREDIMED field centers (*n* = 1000 PREDIMED-Valencia participants with complete data). In addition to the TCF7L2-rs7903146 SNP, we selected nine T2D-SNPs from the list of the 49 SNPs included in the T2D-GRS used in the EPIC-Interact Study [16] (see statistical analysis for the SNP selection) and tested their association with T2D prevalence by obesity strata in our sample. Those SNPs showing significant (or near significance) associations in one of the obesity strata were selected for combination in the corresponding obesity-specific GRS (nobGRS and obGRS). Five SNPs in each score were combined (nobGRS: TCF7L2-rs7903146, PRC1-rs12899811, ZFAND6-rs11634397, CDC123_CAMK1D-rs11257655 and KCNQ1-rs163184; obGRS: ADYC5-rs6798189, IGF2BP2-rs4402960, SLC30A8-rs3802177, KLHDC5-rs10842994, and HMGA2-rs2261181 as indicated in methods). Supplementary Materials Figure S3 shows frequency distribution of nobGRS (A) and obGRS (B) in the whole sample (*n* = 1000). First, we tested the association of these obesity-specific GRS with T2D prevalence in obese and non-obese subjects (Supplementary Materials Table S3). As expected, the nobGRS was significantly associated with T2D in non-obese subjects (*p* = 0.006). No significant association was found for the nobGRS in obese subjects (*p* = 0.535). Likewise, the obGRS was significantly associated with T2D in obese subjects (*p* < 0.001), and not associated in non-obese subjects (*p* = 0.130). We also estimated the predictive value for these obesity-specific GRS for T2D in the whole sample (Supplementary Materials Figure S4) and by obesity strata (Figure 3). When we consider the obesity-specific GRS in their specific strata, we observed that the nobGRS had greater and significantly AUC in non-obese (AUC: 0.581; *p* = 0.002) than in obese subjects (AUC: 0.522; *p* = 0.384). Likewise, the obGRS had higher AUC in obese (0.591; *p* = 0.004) than in non-obese subjects (0.531; *p* = 0.239), thus supporting our hypothesis.

### 3.6. Longitudinal Interaction between the TCF7L2-rs7903146 Polymorphism and T2D in BMI

**Figure 4:**
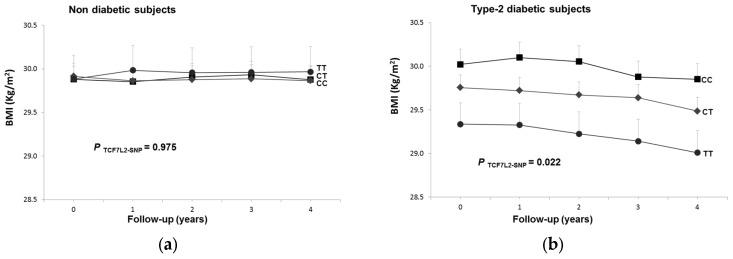
Longitudinal effect of the TCF7L2-rs7903146 polymorphism on BMI over a 4-year follow-up period in *n* = 3141 subjects depending on T2D status: (**a**) non-diabetic; (**b**) T2D subjects. Adjusted means BMI depending on the polymorphism (co-dominant model) and T2D at baseline and 1, 2, 3 and 4 years of follow-up in all subjects having data for all the five measurements were estimated from a repeated-measures ANOVA model with interaction terms adjusted for dietary intervention (MedDiet versus control), sex, age, center, BMI, adherence to the Mediterranean diet (AdMedDiet), smoking, drinking, and physical activity at baseline. Adjusted *p* values for the overall effect of the polymorphism and for the interaction among the polymorphism and T2D, were obtained in the multivariable model.

At baseline, we have observed a shared interactive effect between the FCF7L2-rs7903146 polymorphism and T2D in determining BMI. Here we tested whether this shared interactive effect is also observed longitudinally. We analyzed two periods based on the inclusion of a larger number of participants (at four-year follow-up, with 3141 participants) or on a longer follow-up period (at six-year follow-up, with 1750 participants). Figure 4 shows the association between the TCF7L2-SNP and BMI longitudinally for every year of the four-year follow-up period. We observed a significant heterogeneity (*p*-interaction: 0.041) in non-diabetic (A) and in T2D (B) subjects. In T2D subjects the association between the SNP and lower BMI was detected yearly (*p*-for the average inter-subjects effects: 0.02), whereas in non-diabetic subjects no significant association with BMI was observed over the follow-up period (*p*: 0.975). The later analysis, including participants with six years of follow-up, confirmed these results (Supplementary Materials Figure S5).

## 4. Discussion

Combining longitudinal and cross-sectional analyses in a well-characterized population [30], we have obtained new epidemiological evidence to unravel the complex relationship between the TCF7L2-rs7903146 polymorphism, obesity, and T2D. At the population level, we have obtained consistent results showing that, on the one hand, the TCF7L2-rs7903146 polymorphism significantly interacts with obesity to determine the prevalence and incidence of T2D, and, on the other, that T2D interacts with the TCF7L2-rs7903146 in BMI both at baseline and prospectively. As these three variables are so closely interrelated [1,2] it is very difficult to distinguish cause from effect. According to previous investigations [5,6,8], it seems most plausible that obesity status interacts with the TCF7L2 gene, prompting the TT risk genotype to associate itself with a higher incidence of T2D in lean subjects; perhaps the effect we observed of the T-allele being associated with lower obesity risk in T2D subjects is a secondary observation to the primary one. However, we cannot discard the real influence of the T-allele on body weight. Despite this SNP being one of the strongest common genetic determinant of T2D yet described [9,10,11,34], there is still much controversy over the molecular mechanisms involved in how this genetic variation of TCF7L2 gene leads to altered biological function [35,36,37,38].

Although some previous studies [5,6,12,13,14] have described that the TCF7L2-rs7903146 polymorphism was more strongly associated with T2D in non-obese subjects, most of these studies have been retrospective and prone to potential bias. Only one study has prospectively and specifically analyzed the interaction between the TCF7L2-rs7903146 SNP and BMI in determining T2D incidence [15]. However, this study included only men. Thus, our study, including both men and women, is the first prospective report showing that at the population level (analyzing both men and women) the TCF7L2 polymorphism is more associated with T2D incidence in non-obese subjects. Our results support and extend the findings, adding more prospective evidence to the heterogeneity in the association. Recent prospective studies [16,17,18,19] have analyzed the interaction between genetic variants and BMI on T2D using multi-SNP GRS [39] rather than focusing specifically on the TCF7L2-SNP (which was only one of the loci in the GRS). In the EPIC-InterAct study [16], where they analyzed the interaction between each of the SNPs of the GRS and BMI on T2D, despite the fact that they found that the global GRS significantly interacted with BMI, these authors did not find any significant interaction of BMI with the TCF7L2 SNP after correction for multiple comparisons. These results may be due to the fact that they undertook multiple comparisons and the statistical significance was set at *p* < 0.05/343. These results could be considered as a false negative and so clearly show the need to continue investigating this interaction in more depth. Several studies have examined the effect of other T2D-SNPs integrated in GRSs, among them that from Talmud et al. [19], including seven prospective studies and using a T2D 65-SNP GRS. They showed heterogeneity on the effects of the GRS by BMI in determining T2D (the 65-SNP GRS being associated with higher T2D incidence in leaner individuals). Talmud et al. [19] also evaluated the predictive value of the GRS and showed heterogeneity by BMI [19]. In our study, we have found better results for the TCF7L2-rs7903146 in predicting T2D in non-obese than in obese subjects. In addition to the TCF7L2, other genes [8,40,41] could be associated with higher T2D risk in non-obese individuals. Moreover a recent study carried out in Chinese subjects [42] investigated the association between a 25-SNP GRS (global and by SNP) and T2D prevalence in obese and non-obese subjects separately, concluding that some SNP were strongly associated with T2D in non-obese subjects, whereas others were specific for obese subjects. However, the authors did not construct obesity-specific GRS.

An important finding of our study is that, unlike previous studies, we have constructed obesity-specific GRS by combining in the specific score only those SNPs more associated with T2D in the specific obesity strata. Thus we constructed an obGRS (including the T2D-SNPs more associated with T2D in obese subjects) and a nobGRS (including the T2D-SNPs more associated with T2D in non-obese subjects, such as the TCF7L2). As far as we know, this is the first time that the use of obesity-specific GRS has been tested. Our results show that, consistent with our hypothesis, a GRS including the TCF7L2 polymorphism and others in a nobGRS present greater AUC for T2D prevalence in non-obese than in obese subjects. Likewise, the obGRS presented greater AUC for T2D in obese subjects. These results support the desirability of taking stratification by obesity into account on analyzing the genetic influence in T2D. One limitation of our results is that we only have tested these GRS in a subsample of the PREDIMED study (all the subjects in a field center with genetic data available). Additional replication in the whole cohort or in another population, also using T2D incidence, will be needed to establish the concept of obesity-specific GRS. However, our preliminary results are important for this upcoming work and will improve the predictive values of the obesity-specific GRS. Thus, whereas two SNPs may present similar overall significant association with T2D incidence, this significance could be driven in one case by obesity and in the other by leanness, making it inappropriate to combine them in the same GRS.

Moreover, our results will be highly relevant for future nutrigenetic studies, taking into account that currently heterogeneity by obesity is not evaluated when analyzing gene–diet interactions in determining T2D risk. Controversial results have been reported regarding the protective effects of fiber intake depending on the TCF7L2 polymorphism [43]. According to our results, in studies aimed at analyzing gene–diet interactions involving the TCF7L2 polymorphism in the determination of T2D, stratification by obesity status should be needed. Likewise, much more investigation regarding gene–diet interactions in lean subjects with the high-risk TCF7L2-rs7903146-genotype is needed to better characterize the potential dietary modulations for T2D prevention.

Overall, these data support the need to carry out analyses stratified by obesity when evaluating genetic associations and gene–diet interactions in determining T2D. This will be key when examining the sensitivity and specificity of genetic analyses to predict T2D risk, as these parameters may vary substantially depending on the obesity status of the person submitted to the test. Along these lines, some studies examining the potential utility of the TCF7L2 gene variant for the prediction of T2D have produced disappointing results [44,45]. Assessing heterogeneity by obesity and other factors may help to fill the gap that exists between genetic discoveries and their practical applications to T2D prevention [46,47].

In terms of obesity as an outcome, our results also suggest an inverse association of the TCF7L2 polymorphism with BMI in T2D subjects. In agreement with us, a meta-analysis of GWAs [28] detected such heterogeneity at the cross-sectional level but did not explore this association longitudinally, with stronger effects in T2D case/control studies (the T2D-risk allele associated with lower BMI) than in population-based studies. As far as we know, our study is the first to show a prospective interaction between the TCF7L2-rs7903146 polymorphism and T2D in determining BMI (up to six years). This is also important as other studies have focused on gene–diet interactions on weight changes involving the TCF7L2 polymorphism [48]. In these studies stratification by T2D status should be considered to reduce the confounding effect of this heterogeneity. Our interaction results will be highly relevant for upcoming nutrigenetic studies focusing on the influence of the TCF7L2 polymorphism on BMI.

## 5. Conclusions

In conclusion, we have comprehensively demonstrated that the TCF7L2-rs7903146 polymorphism interacts with obesity status in determining T2D risk, emphasizing the heterogeneity of genetic variants’ T2D risk prediction. We support stratified analysis by obesity or by T2D, depending on whether the association of the TCF7L2 polymorphism with T2D or with BMI is being investigated. We suggest that taking into account this heterogeneity by obesity will improve the predictive value of this SNP in determining T2D risk. Moreover, this heterogeneity of the TCF7L2-rs7903146 effects on T2D can be extended to other T2D SNPs and we have created for the first time the concept of obesity-specific GRS. We have tested two obesity-specific GRS, showing an increase in the predictive value of the GRS in the corresponding strata. The creation and validation of the proposed obesity-specific GRS, including more SNPs, will be important in the new era of personalized genetic risk prediction for T2D as well as in nutrigenetic studies for more accurate testing of gene–diet interactions.

## Figures and Tables

**Table 1:** Demographic, clinical, lifestyle, and genetic characteristics of the study participants at baseline according to the diabetes status.

	Total (*n* = 7018)		Non-Diabetic Subjects (*n* = 3607)		T2D Subjects (*n* = 3411)		*p*
Age (years)	67.0	±6.2	66.6	±6.1	67.4	±6.3	<0.001
Weight (Kg)	76.8	±11.9	76.7	±11.7	76.9	±12.2	0.476
BMI (Kg/m^2^)	30.0	±3.8	30.1	±3.7	29.9	±4.0	0.042
Waist circumference (cm)	100.4	±10.6	99.7	±10.6	101.2	±10.5	<0.001
Body fat (%)	39.3	±7.4	39.9	±7.2	38.7	±7.7	<0.001
Female sex: *n*, %	4025	(57.4)	2232	(61.9)	1793	(52.6)	<0.001
Current smokers: *n*, %	989	(14.1)	581	(16.1)	408	(12.0)	<0.001
*TCF7L2*-rs7903146: *n*, %							<0.001
CC	2770	(39.5)	1612	(44.7)	1158	(33.9)	
CT	3249	(46.3)	1569	(43.5)	1680	(49.3)	
TT	999	(14.2)	426	(11.8)	573	(16.8)	
Intervention groups: *n*, %							0.059
MedDiet + EVOO	2411	(34.4)	1204	(33.4)	1207	(35.4)	
MedDiet + nuts	2316	(33.0)	1235	(34.2)	1081	(31.7)	
Control group	2291	(32.6)	1168	(32.4)	1123	(32.9)	
Energy intake (kcal/day)	2276	±607	2322	±603	2228	±607	<0.001
Total fat (% energy)	39.2	±6.8	38.5	±6.5	39.9	±7.0	<0.001
Saturated fat (% energy)	10.0	±2.3	9.7	±2.2	10.2	±2.3	<0.001
MUFA (% energy)	19.5	±4.6	19.2	±4.3	19.7	±4.8	<0.001
Carbohydrates (% energy)	41.9	±7.2	42.8	±6.9	40.9	±7.3	<0.001
Adherence to the MedDiet	8.7	±2.0	8.7	±2.0	8.6	±2.0	0.003
Alcohol consumption (g/day)	8.4	±14.2	9.1	±14.8	7.6	±13.5	<0.001
Physical activity (MET.min/day)	231.6	±240.4	225.5	±226.8	238.0	±253.8	0.030
SBP (mm·Hg)	149.3	±20.8	149.0	±20.6	149.7	±21.0	0.187
DBP (mm·Hg)	83.4	±11.0	84.5	±11.0	82.2	±10.9	<0.001
Total cholesterol (mg/dL)	211.0	±39.4	220.0	±39.8	201.4	±36.6	<0.001
LDL-C (mg/dL)	130.3	±35.1	137.9	±36.2	122.1	±31.8	<0.001
HDL-C (mg/dL)	53.8	±14.1	55.8	±14.6	51.7	±13.2	<0.001
Triglycerides (mg/dL)	137.4	±79.7	132.6	±73.9	142.4	±85.2	<0.001
Fasting glucose (mg/dL)	122.2	±41.6	98.2	±16.4	147.4	±45.1	<0.001

Values are mean ± SD for continuous variables and number (%) for categorical variables. T2D indicates Type 2 diabetes. BMI indicates body mass index, MUFA, Monounsaturated fatty acids; MedDiet, Mediterranean diet; EVOO, extra virgin olive oil, SPB: Systolic blood pressure, DBP: Diastolic blood pressure. *p*: *p*-value for the comparisons (means or %) between non-diabetic and type 2 diabetic subjects.

**Table 2:** Association between the TCF7L2-rs7903146 polymorphism and prevalence of T2D depending on the obesity status at baseline. Stratified logistic regression analysis.

	Non-Obese				*p* ^3^ for Interaction	Obese			
	*n*	OR	95% CI	*p*-Value	Genotype × Obesity	*n*	OR	95% CI	*p*-Value
Model 1 ^1^									
*TCF7L2*					0.002				
CC	1424	1.00	(reference)			1346	1.00	(reference)	
CT	1742	1.79	(1.55–2.08)	5.5 × 10^−15^		1507	1.28	(1.10–1.49)	0.0012
TT	573	2.32	(1.90–2.85)	5.4 × 10^−16^		426	1.51	(1.21–1.89)	0.0003
		*p* ^4^: 2.5 × 10^−20^					*p* ^4^: 0.0002		
Model 2 ^2^									
*TCF7L2*									
CC		1.00	(reference)				1.00	(reference)	
CT		1.78	(1.54–2.09)	1.4 × 10^−14^	0.003		1.27	(1.09–1.48)	0.0020
TT		2.26	(1.84–2.78)	1.6 × 10^−14^			1.53	(1.22–1.92)	0.0002
		*p* ^4^: 4.3 × 10^−19^					*p* ^4^: 0.00017		

^1^ Model 1: adjusted for sex, age, and field center; ^2^ Model 2: adjusted for sex, age, field center, total energy intake, adherence to the Mediterranean diet, alcohol intake, smoking, physical activity, dyslipidemia, and hypertension; ^3^ *p*-value obtained for the interaction term between the TCF7L2 genotype and obesity in the corresponding multivariable logistic regression model; ^4^ *p*-value obtained for the global TCF7L2 polymorphism in the multivariable logistic regression model.

**Table 3:** Association between the TCF7L2-rs7903146 polymorphism and obesity depending on T2D status. Stratified logistic regression analysis.

	Non-Diabetic				*p* ^3^ for Interaction	T2D Subjects			
	*n*	OR	95% CI	*p*-Value	Genotype × T2D	*n*	OR	95% CI	*p*-Value
Model 1 ^1^									
*TCF7L2*					0.008				
CC	1612	1.00	(reference)			1158	1.00	(reference)	
CT	1569	1.05	(0.91–1.20)	0.544		1680	0.78	(0.67–0.91)	0.001
TT	426	0.96	(0.77–1.19)	0.694		573	0.64	(0.52–0.78)	2.0 × 10^−5^
		*p* ^4^: 0.685					*p* ^4^: 3.90 × 10^−5^		
Model 2 ^2^									
*TCF7L2*									
CC		1.00	(reference)				1.00	(reference)	
CT		1.05	(0.91–1.21)	0.529	0.014		0.77	(0.66–0.90)	0.001
TT		0.93	(0.74–1.16)	0.493			0.63	(0.51–0.78)	1.6 × 10^−5^
		*p* ^4^: 0.528					*p* ^4^: 3.02 × 10^−5^		

^1^ Model 1: adjusted for sex, age, and field center; ^2^ Model 2: adjusted for sex, age, field center, total energy intake, adherence to the Mediterranean diet, alcohol intake, smoking, physical activity, dyslipidemia, and hypertension; ^3^ *p*-value obtained for the interaction term between the TCF7L2 genotype and type-2 diabetes in the corresponding multivariable logistic regression model; ^4^ *p*-value obtained for the global TCF7L2 polymorphism in the multivariable logistic regression model.

**Table 4:** Incidence and hazard ratios (HR) for T2D depending on the TCF7L2-rs7903146 polymorphism and stratified by obesity after 5.7 years of median follow-up.

	**Obese Subjects ^1^ (*n* = 1693)**									
	**Cases**	**Non-Cases**	**Person-Years**	**Incidence Rate ^4^**	***Model 1*^2^**			***Model 2*^3^**		
					**HR**	**95% CI**	***p*-Value**	**HR**	**95% CI**	***p*-Value**
*TCF7L2 genotypes*							0.960			0.965
CC	73	677	4117.5	17.7	1.00	(reference)		1.00	(reference)	
CT	79	669	4203.8	18.8	1.04	(0.76–1.44)	0.777	1.05	(0.76–1.46)	0.750
TT	21	174	1094.0	19.2	1.01	(0.62–1.65)	0.957	1.01	(0.61–1.66)	0.979
	**Non-Obese Subjects (*n* = 1904)**									
					***Model 1*^2^**			***Model 2*^3^**		
*TCF7L2 genotypes*							0.035			0.045
CC	56	803	4819.0	11.6	1.00	(reference)		1.00	(reference)	
CT	57	758	4661.8	12.2	1.08	(0.75–1.57)	0.671	1.14	(0.79–1.66)	0.531
TT	26	204	1285.7	20.2	1.82	(1.14–2.92)	0.012	1.81	(1.13–2.92)	0.013

^1^ Obesity: BMI ≥30 kg/m^2^; ^2^ Model 1: Adjusted for sex, age, field center, and dietary intervention group; ^3^ Model 2: Adjusted for variables in model 1 plus total energy intake, adherence to the Mediterranean diet, alcohol intake, smoking, physical activity, dyslipidemia, and hypertension at baseline; ^4^ Crude incidence rates are expressed per 1000 person-years of follow-up.

## Supplementary Materials

The following are available online at http://www.mdpi.com/2072-6643/8/12/793/s1.
