# Supplementary material for: Polymorphism of the Transcription Factor 7-Like 2 Gene (TCF7L2) Interacts with Obesity on Type-2 Diabetes in the PREDIMED Study Emphasizing the Heterogeneity of Genetic Variants in Type-2 Diabetes Risk Prediction: Time for Obesity-Specific Genetic Risk Scores
Source: Nutrients. 2016 Dec 6;8(12):793. doi: 10.3390/nu8120793 (PMC5188448; doi:10.3390/nu8120793)
Supplement: Supplementary file 1 [file nutrients-08-00793-s001.docx]

Supplementary Materials: Polymorphism of the Transcription Factor 7-Like 2 Gene (TCF7L2) Interacts with Obesity on Type-2 Diabetes in the PREDIMED Study Emphasizing the Heterogeneity of Genetic Variants in Type-2 Diabetes Risk Prediction: Time for Obesity-Specific Genetic Risk Scores

Dolores Corella, Oscar Coltell, Jose V. Sorlí, Ramón Estruch, Laura Quiles, Miguel Ángel Martínez-González, Jordi Salas-Salvadó, Olga Castañer, Fernando Arós, Manuel Ortega-Calvo, Lluís Serra-Majem, Enrique Gómez-Gracia, Olga Portolés, Miquel Fiol, Javier Díez Espino, Josep Basora, Montserrat Fitó, Emilio Ros and José M. Ordovás


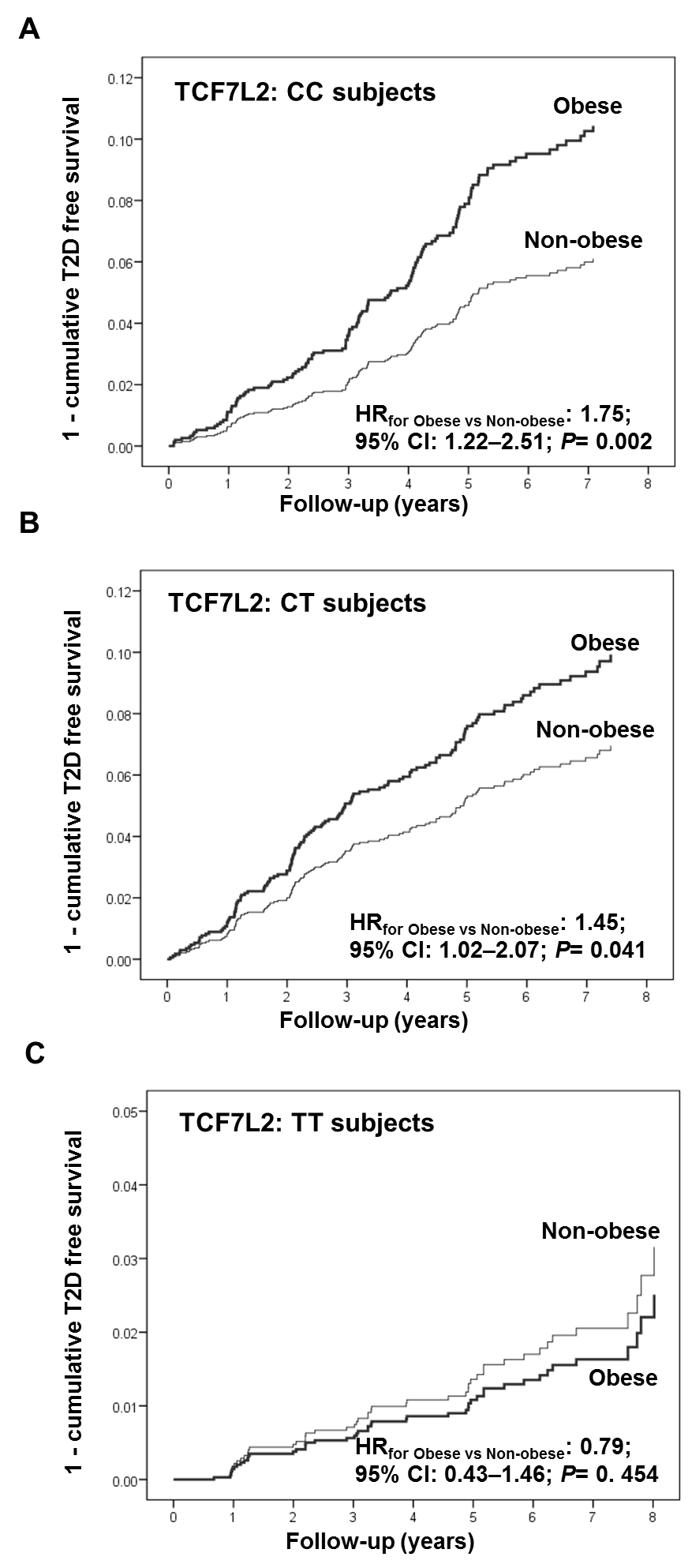


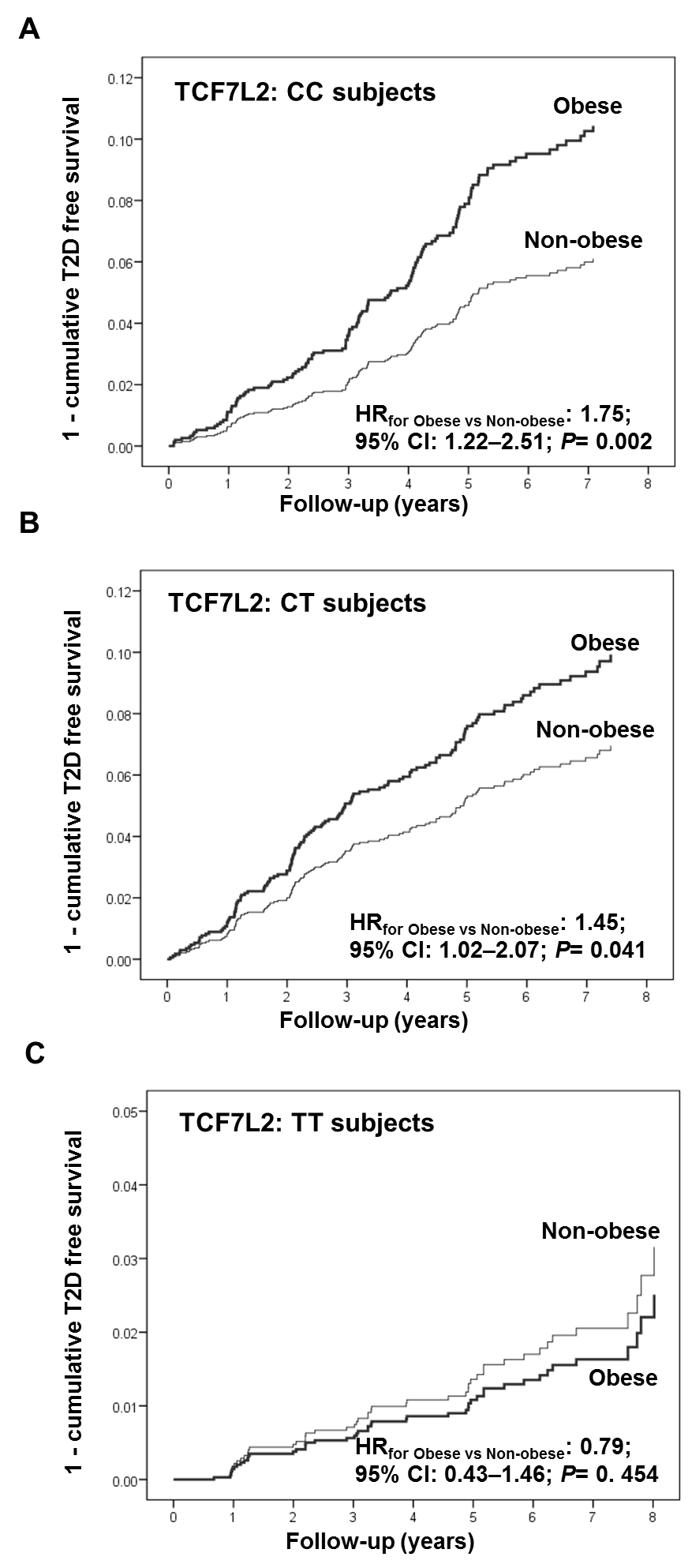


**Figure S1.** One minus the cumulative type-2 diabetes-free survival by TCF7L2-rs7903146 genotypes in non-diabetic subjects at baseline (*n* = 3607) depending of the obesity status and the TCF7L2-rs7903146 polymorphism (**A**) CC; (**B**) CT; and (**C**) TT subjects. Cox regression models with outcome of type-2 diabetes incidence were adjusted for sex, age, center, intervention group, alcohol, smoking, total energy intake and adherence to the Mediterranean diet, physical activity, smoking, drinking, dyslipemia, and hypertension at baseline. HR and 95% CI were obtained in the multivariable adjusted model. The *p*-values for obese vs. non-obese and for the corresponding genotypes were obtained in the multivariable adjusted models.


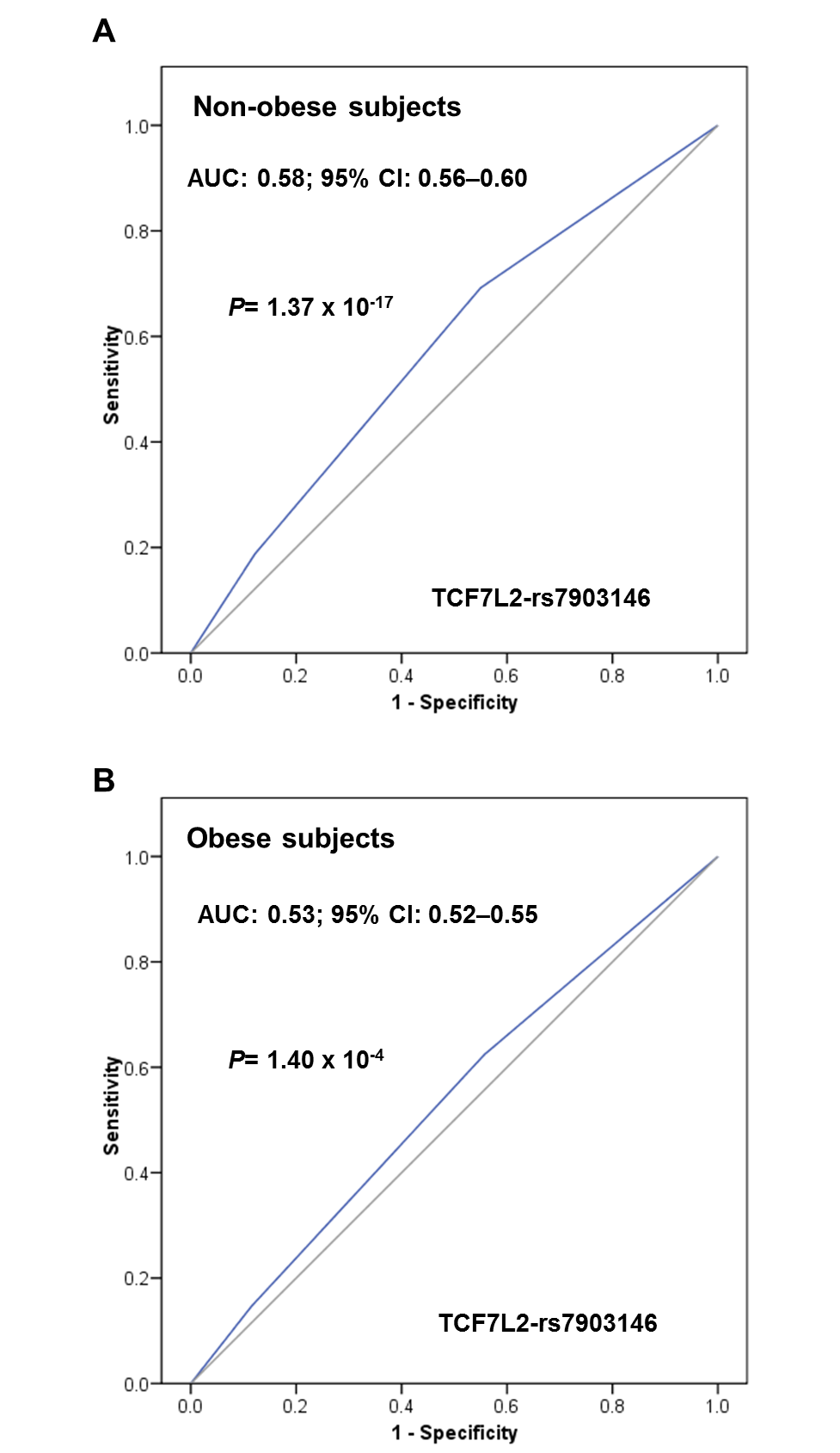


**Figure S2.** Receiver operating curve (ROC) of the TCF7L2-rs7903146 polymorphism (additive) for predicting type-2 diabetes (prevalent) at baseline in (**A**) non-obese and (**B**) obese subjects in the whole PREDIMED population (*n* = 7018). Areas under the curves (AUC) and *p*-values are indicated. The straight line represents the ROC expected by chance only.


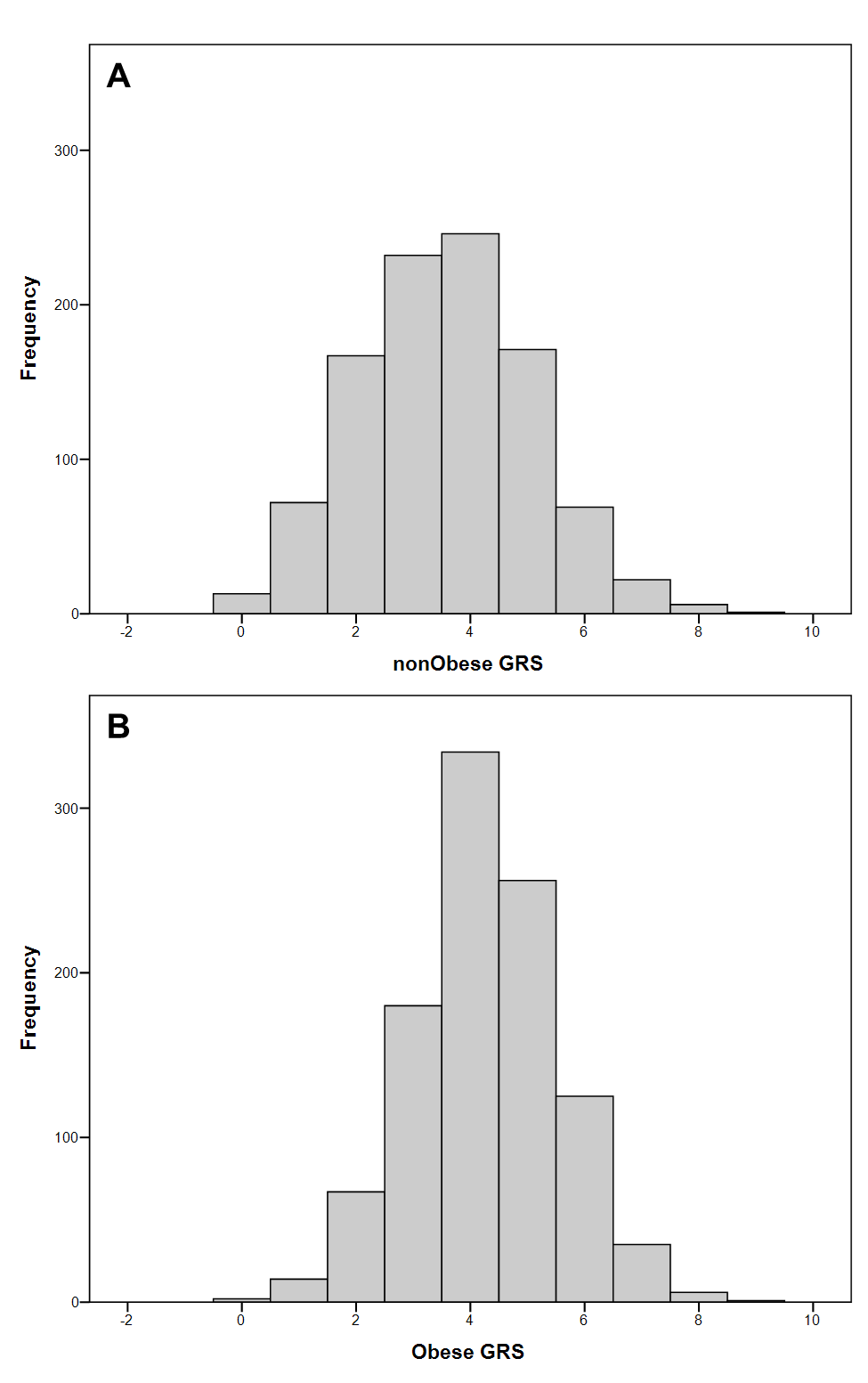


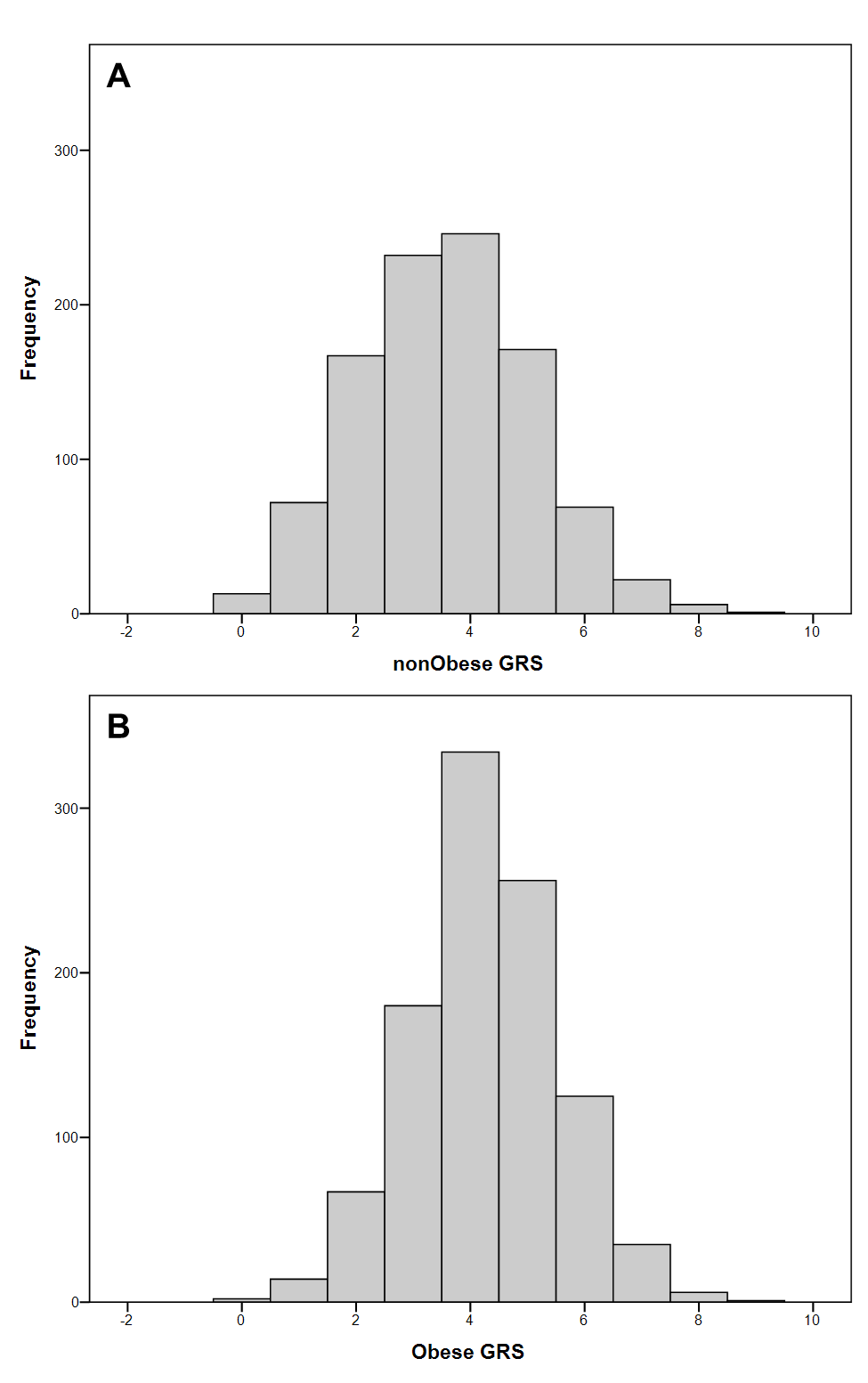


**Figure S3.** Distribution of the unweighted genetic risk scores (GRS) in the PREDIMED-Valencia participants (*n* = 1000): (**A**) distribution of the obese-GRS including the following SNPs: ADYC5-rs6798189, IGF2BP2-rs4402960, SLC30A8-rs3802177, KLHDC5-rs10842994, and HMGA2-rs2261181; (**B**) distribution of the non-obese-GRS including the following SNPs: TCF7L2-rs7903146, PRC1-rs12899811, ZFAND6-rs11634397, CDC123_CAMK1D-rs11257655, and KCNQ1-rs163184.


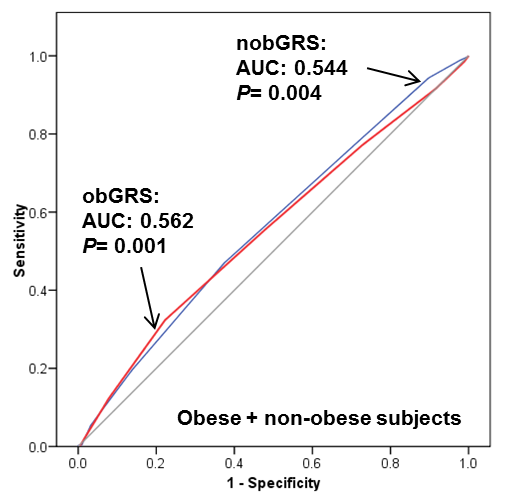


**Figure S4.** Receiver operating curves (ROC) of the two Genetic Risk Scores (GRS) to predict type-2 diabetes (prevalent) in the whole sample (obese + non-obese subjects), at baseline in the PREDIMED-Valencia participants (*n* = 1000). One GRS is including type-2 diabetes-SNPs (ADYC5-rs6798189, IGF2BP2-rs4402960, SLC30A8-rs3802177, KLHDC5-rs10842994, and HMGA2-rs2261181) more associated in obese subjects (obGRS); and the other is including type-2 diabetes-SNPs (TCF7L2-rs7903146, PRC1-rs12899811, ZFAND6-rs11634397, CDC123_CAMK1D- rs11257655, and KCNQ1-rs163184) more associated in non-obese subjects (nobGRS).

| 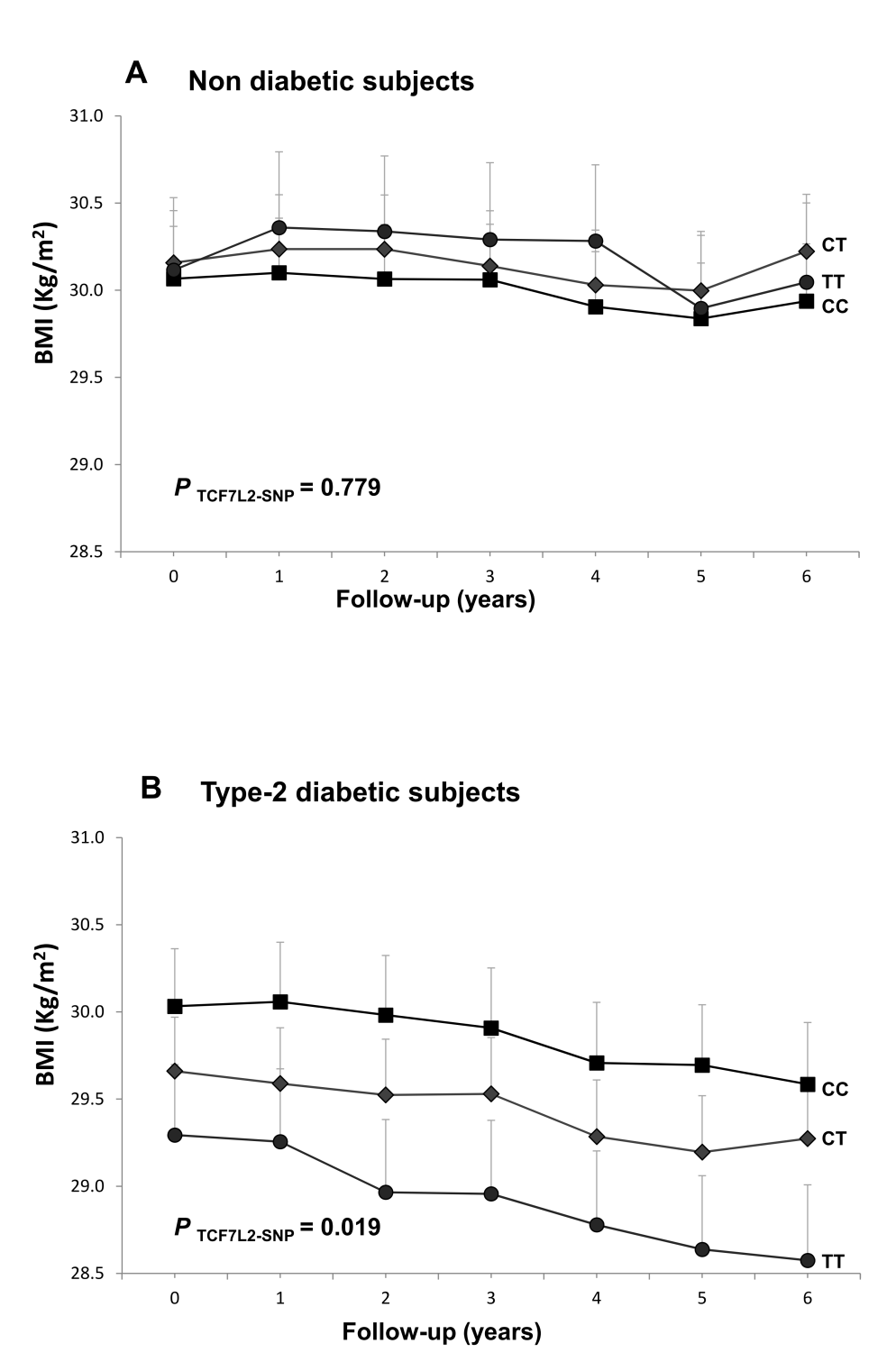 |
| --- |
| 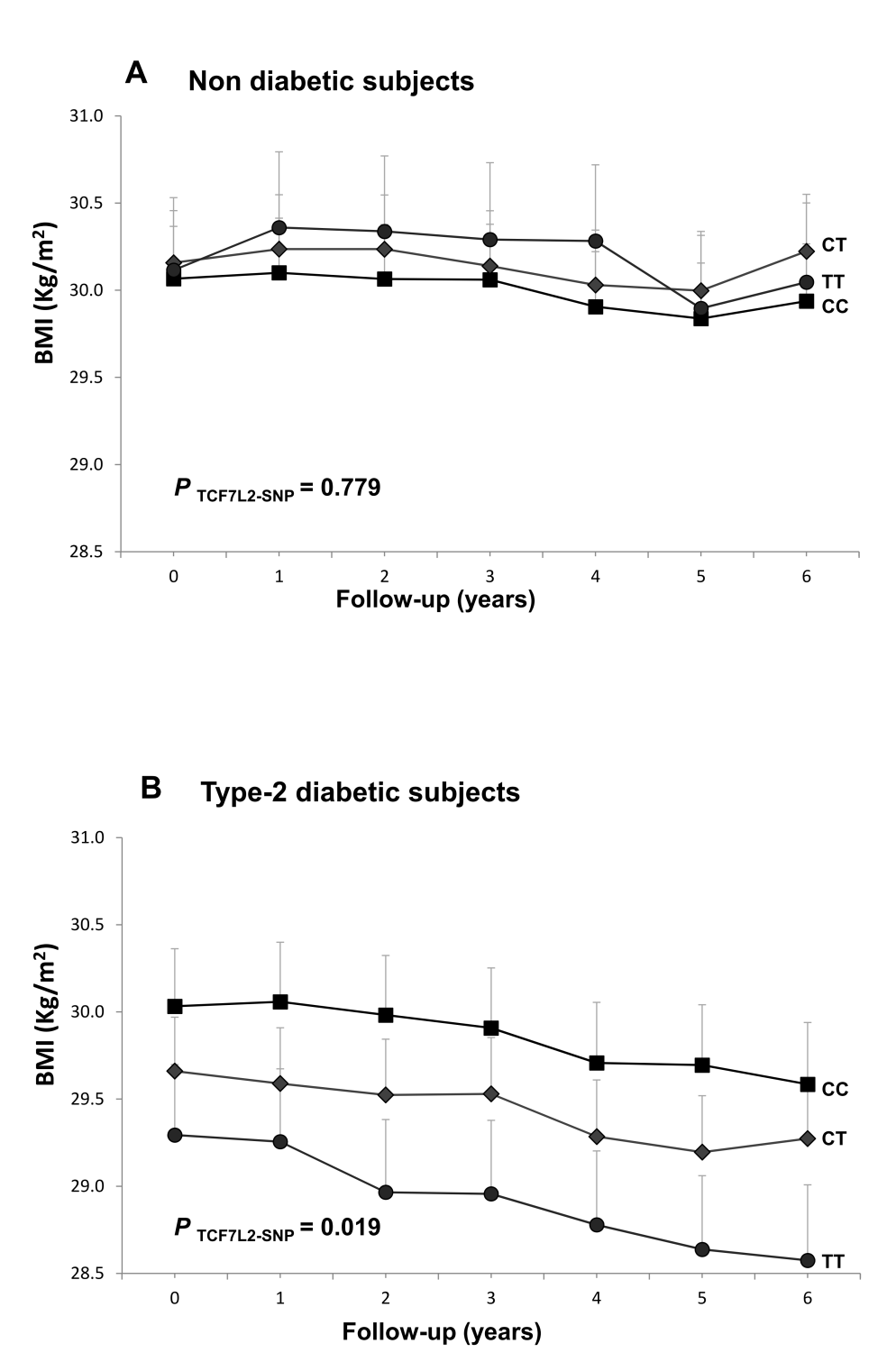 |

**Figure S5.** Longitudinal effect of the TCF7L2-rs7903146 polymorphism on BMI over a 6-year follow-up period in *n* = 1750 subjects depending on type-2 diabetes status: (**A**) non-diabetic; (**B**) type-2 diabetes subjects. Adjusted means BMI depending on the polymorphism (co-dominant model) and type-2 diabetes at baseline and 1, 2, 3, 4, 5, and 6 years of follow-up in all subjects having data for all the seven measurements were estimated from a repeated-measures ANOVA model with interaction terms adjusted for dietary intervention (MedDiet versus control), sex, age, center, BMI, adherence to the Mediterranean diet, smoking, drinking, and physical activity at baseline. Adjusted P values for the overall effect of the polymorphism and for the interaction among the polymorphism and type-2 diabetes were obtained in the multivariable model.

**Table S1.** Association between the TCF7L2-rs7903146 polymorphism and obesity-related traits at baseline.

|  | ***TCF7L2 Genotypes*** | | | | | |  | |
| --- | --- | --- | --- | --- | --- | --- | --- | --- |
|  | **CC** | | **CT** | | **TT** | |  |  |
|  | **(*n* = 2770)** | | **(*n* = 3249)** | | **(*n* = 999)** | |  |  |
| **Parameter** | **Mean** | **SD** | **Mean** | **SD** | **Mean** | **SD** | ***p* ^1^** | ***p* ^2^** |
| Age (years) | 67.0 | (6.1) | 67.0 | (6.2) | 66.7 | (6.4) | 0.392 |  |
| Weight (kg) | 77.0 | (12.0) | 76.7 | (11.9) | 76.4 | (11.5) | 0.273 | 0.078 |
| BMI (kg/m^2^) | 30.2 | (3.9) | 29.9 | (3.8) | 29.6 | (3.8) | <0.001 | 3.5 × 10^−4^ |
| Waist circumference (cm) | 100.7 | (10.7) | 100.2 | (10.6) | 100.2 | (10.0) | 0.147 | 0.007 |
| Waist-to-height ratio (in %) | 63.2 | (6.7) | 62.7 | (6.7) | 62.5 | (6.4) | 0.007 | 0.001 |
| Body fat (%) | 39.6 | (7.4) | 39.2 | (7.4) | 38.7 | (7.6) | 0.005 | 3.8 × 10^−4^ |
| Obesity *: *n* (%) | 1346 | (48.6) | 1507 | (46.4) | 426 | (42.6) | 0.005 |  |
| Obesity * risk (OR and 95% CI) | Ref. | | 0.92 (0.83–1.01) | | 0.79 (0.68–0.91) | | 0.005 | 0.002 |

*: Values are means and standard deviations (SD) or *n* (%) or odds ratio, OR and 95% confidence intervals (95% CI). ^1^ Unadjusted *p* values for mean comparison among genotypes. ^2^ *p* values adjusted for sex, age, center, diabetes, total energy intake, adherence to the Mediterranean diet, alcohol consumption, smoking, physical activity, dyslipidemia and hypertension. *: Obesity: BMI ≥ 30 kg/m^2^.

**Table S2.** Sensitivity, specificity and predictive value for the TCF7L2-rs7903146 polymorphism for type-2 diabetes incidence in non-diabetic subjects at baseline in a recessive model. Analysis by obesity strata.

|  | **Non-obese Subjects (*n* = 1904)** | | | **Obese Subjects (*n* = 1693)** | | | | |
| --- | --- | --- | --- | --- | --- | --- | --- | --- |
|  | **TD2 Incidence** | | | **TD2 Incidence** | | | | |
|  | **Yes (*n*)** | **No (*n*)** | ***p*-Value** | **Yes (*n*)** | | **No (*n*)** | ***p* Value** |  |
| ***Test (TCF7L2)*** |  |  | 0.013 |  | |  | 0.787 |  |
| Positive (TT) | 26 | 204 |  | 21 | | 174 |  |  |
| Negative (CC or CT) | 113 | 1561 |  | 152 | | 1346 |  |  |
|  | **Non-Obese** | | | **Obese Subjects** | | | | |
| **Parameters** | **Value** | **95% CI** | | **Value** | **95% CI** | | |  |
| Sensitivity *(%)* | 18.71 | 12.60–26.19 | | 12.14 | 7.67–17.96 | | |  |
| Specificity *(%)* | 88.44 | 86.86–89.90 | | 88.55 | 86.84–90.11 | | |  |
| Positive likelihood ratio | 1.62 | 1.12–2.34 | | 1.06 | 0.69–1.62 | | |  |
| Negative likelihood ratio | 0.92 | 0.85–1.00 | | 0.99 | 0.94–1.05 | | |  |
| Disease prevalence (estimated) *(%)* | 7.30 | 6.17–8.56 | | 10.22 | 8.82–11.76 | | |  |
| Positive predictive value *(%)* | 11.30 | 7.52–16.12 | | 10.77 | 6.79–15.99 | | |  |
| Negative predictive value *(%)* | 93.25 | 91.94–94.40 | | 89.85 | 88.21–91.34 | | |  |

**Sensitivity**: probability that a test result will be positive when the disease is present (true positive rate). **Specificity**: probability that a test result will be negative when the disease is not present (true negative rate). **Positive likelihood ratio**: ratio between the probability of a positive test result given the presence of the disease and the probability of a positive test result given the absence of the disease. **Negative likelihood ratio**: ratio between the probability of a negative test result given the presence of the disease and the probability of a negative test result given the absence of the disease. **Positive predictive value**: probability that the disease is present when the test is positive. **Negative predictive value**: probability that the disease is not present when the test is negative. *p*-value obtained in the Chi-square test in non-obese and obese subjects respectively.

**Table S3.** Association between the obesity-specific genetic risk score (GRS) and type-2 diabetes risk (prevalence OR and 95% CI) at baseline in the whole population and by obesity strata ^1^.

|  | **Whole Population** | | | | **Obese Subjects** | | | | **Non-Obese Subjects** | | | |
| --- | --- | --- | --- | --- | --- | --- | --- | --- | --- | --- | --- | --- |
|  | ***n*** | **OR** | **95% CI** | ***p* Value** | ***n*** | **OR** | **95% CI** | ***p* Value** | ***n*** | **OR** | **95% CI** | ***p* Value** |
| Obese GRS ^2^ | 1000 | 1.21 (1.09–1.33) | | <0.001 | 507 | 1.29 (1.13–1.49) | | <0.001 | 493 | 1.12 (0.97–1.28) | | 0.130 |
| Non–obese GRS ^3^ | 1000 | 1.10 (1.02–1.20) | | 0.020 | 507 | 1.04 (0.92–1.17) | | 0.535 | 493 | 1.18 (1.05–1.33) | | 0.006 |

^1^ The whole population is PREDIMED-Valencia participants. Models adjusted for sex, age and obesity. GRS were analyzed as continous variables. ^2^ Obese GRS includes the following SNPs: ADYC5-rs6798189, GF2BP2_rs4402960, SLC30A8_rs3802177, KLHDC5_rs10842994 and HMGA2-rs2261181 (unweighted). ^3^ Non-obese GRS includes the following SNPs: TCF7L2-rs7903146, PRC1-rs12899811, ZFAND6-rs11634397, CDC123_CAMK1D-rs11257655 and KCNQ1-rs163184 (unweighted).
